# Realities and challenges of breastfeeding policy in the context of HIV: a qualitative study on community perspectives on facilitators and barriers related to breastfeeding among HIV positive mothers in Baringo County, Kenya

**DOI:** 10.1186/s13006-021-00385-1

**Published:** 2021-05-08

**Authors:** Betty Mogesi Samburu, Judith Kimiywe, Sera Lewise Young, Frederick Murunga Wekesah, Milka Njeri Wanjohi, Peter Muriuki, Nyovani Janet Madise, Paula L. Griffiths, Elizabeth W. Kimani-Murage

**Affiliations:** 1 United Nations Children’s Fund (UNICEF), Nairobi, Kenya; 2 Department of Foods, Nutrition and Dietetics, Kenyatta University, Nairobi, Kenya; 3 Institute of Policy Research, Northwestern University, Evanston, USA; 4 Maternal and Child Wellbeing Unit, African Population and Health Research Center, Nairobi, Kenya; 5 Julius Global Health, Julius Center for Health Sciences and Primary Care, University Medical Center Utrecht, Utrecht University, Utrecht, the Netherlands; 6 Institute of Global Health Equity Education, University of Global Health Equity, Kigali, Rwanda; 7 African Institute for Development Policy, Lilongwe, Malawi; 8 School of Sports, Exercise and Health Sciences, Loughborough University, Loughborough, UK; 9 MRC/WITS Developmental Pathways for Health Research Unit (DPHRU), School of Clinical Medicine, Faculty of Health Sciences, University of the Witwatersrand, Johannesburg, South Africa; 10 Wellcome Trust, London, UK; 11 Human Nutrition, School of Medicine, Dentistry and Nursing, College of Medical, Veterinary & Life Sciences, University of Glasgow, Glasgow, G31 2ER UK; 12 Stellenbosch Institute for Advanced Study (STIAS), Wallenberg Research Centre, Stellenbosch University, Stellenbosch, South Africa; 13 International Health Institute, Brown University School of Public Health, Providence, USA

**Keywords:** Exclusive breastfeeding, Continued breastfeeding, HIV, Facilitators, Barriers, Policy, Kenya

## Abstract

**Background:**

Although recent policies have sought to increase the rates of exclusive breastfeeding (EBF) and continued breastfeeding for HIV exposed infants, few programs have considered the multiple social and cultural barriers to the practice. Therefore, to generate evidence for exclusive and continued breastfeeding policies in Kenya, we examined community perspectives on the facilitators and barriers in adherence to EBF for the HIV positive mothers.

**Methods:**

Qualitative research was conducted in Koibatek, a sub-County in Baringo County Kenya, in August 2014 among 205 respondents. A total of 14 focus group discussions (*n* = 177), 14 In-depth Interviews and 16 key informant interviews were conducted. Transcribed data was analyzed thematically. NVivo version 10.0 computer qualitative software program was used to manage and facilitate the analysis.

**Results:**

Facilitators to exclusive breastfeeding were perceived to include counselling at the health facility, desire to have a healthy baby, use of antiretroviral drugs and health benefits associated with breastmilk. Barriers to EBF included poor dissemination of policies, knowledge gap, misinterpretation of EBF, inadequate counselling, attitude of mother and health workers due to fear of vertical HIV transmission, stigma related to misconception and misinformation that EBF is only compulsory for HIV positive mothers, stigma related to HIV and disclosure, social pressure, lack of male involvement, cultural practices and traditions, employment, food insecurity.

**Conclusions:**

There are multiple facilitators and barriers of optimal breastfeeding that needs a holistic approach to interventions aimed at achieving elimination of mother to child transmission. Extension of infant feeding support in the context of HIV to the community while building on existing interventions such as the Baby Friendly Community Initiative is key to providing confidential support services for the additional needs faced by HIV positive mothers.

## Background

Human milk is the only best source of nutrition for infants in the first 6 months of life [1]. Suboptimal breastfeeding results in up to 823,000 deaths annually in low- and middle-income countries (LMICs) [2]. Children who are not breastfed exclusively have a five and a seven fold increased risk of death due to pneumonia and diarrhea respectively [3, 4]. Long-term health benefits of breastfeeding include reduced risk of obesity, allergies, heart disease and diabetes in adulthood [5, 6].

While breastfeeding is recommended as the optimal and safest way to feed an infant, it has remained a challenge to mothers who are infected with HIV virus. This has led to evolvement of several recommendations overtime to ensure that the baby gets the advantages of breast milk at the same time making it safer to prevent infection from the virus [7–9]. In the late nineties, the World Health Organization (WHO) recommendations on infant feeding in the context of HIV were strongly geared towards the support of replacement feeding and avoiding breastfeeding [10]. However, increased infant mortality from diarrheal disease and respiratory infections for children who were not breastfed led to review of the recommendations [7, 11, 12]. It was concluded that breastfeeding provides optimal nutrition and prevents diarrhea, and a recommendation to increase breastfeeding duration, regardless of maternal HIV-status was proposed [12].

Further evidence revealed that widespread use of anteritroviral (ARV) drugs drastically reduced the risk of mother-to-child transmission (MTCT) rates to < 2%. The PROMISE trial in South Africa found the transmission risk when the mother was on combination antiretroviral therapy (ART) to be as low as 0.3% at 6 months, and 0.7% at 12 months [13]. A systematic review and meta-analysis conducted on postnatal HIV transmission in breastfed infants of HIV infected women on ART found significant reduced postnatal transmission rates of 1.08 (95% CI 0.32, 1.85) at 6 months and 2.93 (95% CI 0.68, 5.18) at 12 months [14].

Based on several studies, WHO and Kenyan guidelines were revised in the year 2009 and 2012 consecutively, to recommend exclusive breastfeeding for HIV-exposed infants and continued breastfeeding in addition to optimal complementary feeding to just 1 year while adhering to anteritroviral drugs [15, 16]. In the year 2016, WHO further revised the guidelines to recommend EBF for the first 6 months of life and continued breastfeeding in addition to adequate, appropriate and safe complementary feeding for 2 years or beyond for both HIV-unexposed and –exposed infants [9]. These recommendations were later adopted for Kenya in 2018, where breastfeeding duration was extended to 2 years or beyond while the mother was put on lifelong ART uninterrupted or option B+ [8]. Current evidence from research, while taking into consideration the feasibility and cost implications are clear that for the global majority breastfeeding and antiretroviral (ARVs) will, most likely, give infants born to mothers known to be HIV-infected, the greatest chance of HIV-free survival.

Despite the guidelines and well-known advantages of breastfeeding, the rates of EBF and continued breastfeeding for HIV exposed infants still remain low in Kenya. Approximately 59% of HIV-exposed infants less than 6 months old are exclusively breastfed, and only 32% are breastfed up to 1 year [17]. At the same time, EBF rates in the general population are still low at 61% overall [18]. In Baringo County where this study was conducted, EBF rates is at 36.5% [18, 19]. With these low rates of breastfeeding even among HIV positive mothers, there is need to gather evidence on how such mothers would be supported to adhere to optimal feeding recommendations.

Infant feeding is influenced by a combination of factors at different levels such as individual related to personal obstacles e.g., discouragement, worry about the baby, self-efficacy, expectations, beliefs and attitudes, stigma, lack of knowledge on how to overcome physical and social issues. For example, Some studies have documented a mother’s worry about not having enough milk [20]. Failure to disclose HIV status to family members due to fear of stigma and discrimination have impacted exclusive breastfeeding [21, 22].

Interpersonal setting including social support systems such as family, friends, peers, co-workers, customs and traditions can influence individual behaviors. Culture is known to impact breastfeeding such that mothers who do not follow traditional norms risk exclusion by family members [23]. At the community level, relationship among organizations, institutions and informational networks for example community, healthcare setting, workplace, physical, social and emotional support during pregnancy and breastfeeding period may affect infant feeding. Organizations or social institutions with rules and regulations for operation may affect how well infant feeding is practiced e.g. access to healthcare services, restrictive policies or lack of policies [24–26].

Policies developed at the national level often do not consider the above challenges [27–29]. Previous studies have focused on medical or nutritional factors as consequences for breastfeeding infants born to HIV positive mothers while women’s attitudes, experiences and support they receive from those surrounding them have been ignored. While trying to adhere to EBF and continued breastfeeding for 2 years or beyond, individual, societal or policies that facilitate or hinder have been neglected. Therefore, we sought to explore community perspective on the facilitators and barriers faced by HIV positive mothers in their communities regarding adhering to the national recommendations for breastfeeding in the context of HIV. We sought to gain rounded community perspectives on this topic both from HIV positive mothers and other community members to gain a holistic community perspective on this topic. Employing qualitative participatory methodology enabled the researcher obtain findings that would contribute to community informed ways of promoting safe breastfeeding practices for HIV positive mothers and offer insights on how these mothers can be supported when making informed infant feeding decisions.

## Methods

### Study area and period

The study was conducted in Koibatek one of the six sub-counties in Baringo County, Kenya between August to September 2014. The sub-County is mainly inhabited by the Tugen, a Kalenjin sub-tribe whose main activity is mixed farming. There are also other small tribes like the Nubian community who inhabit a small part of town. In the year between 2014 to 2015 when this study was conducted, Koibatek sub-County had a total of 5206 expected pregnancies out of whom 129 were identified as HIV positive. It ranked 20th out of 47 counties in Kenya with the highest positivity rate at first PCR of 6.8% as compared to the national average of 6.4% [30]. The target is to reduce this average rate to 2.6% by 2021. At the same point in time it ranked 9th out of 47 in terms of missed opportunities for mothers to receive ARV where only 30% of mothers had access to ARV prophylaxis [30, 31]. In the same period only 41% of pregnant mothers attended the recommended four antenatal visits while only 38% of HIV positive pregnant women delivered in a health facility [31]. Baringo has seen small improvement in HIV infection reduction of less than 50% among children measured per 100,000 live births, as compared to other counties that have achieved a reduction of greater than 50% in the period between 2013 to 2015 [30].

### Study design

This was a qualitative study conducted as part of an ongoing community cluster randomized trial study of the Baby Friendly Community Initiative (BFCI) implemented between October 2014 and July 2017 [32, 33]. The BFCI study sought to test the feasibility and effectiveness of BFCI in promoting infant and young child feeding practices where 13 community units (CUs) were randomized six into BFCI intervention and seven to control. The intervention involved counseling and support on breastfeeding. The findings for qualitative formative study were used to inform intervention and design.

### Study population

HIV infected or un-infected mother who had a child under age 2 years, community leaders, religious leaders, traditional birth attendants, herbalist, health administrators, community health volunteers (CHVs), healthcare providers, mothers, fathers, grandmothers who were residing in Koibatek for at least 6 months were included in the study.

### Sample size and sampling strategy

A total of 44 interviews were conducted, with a total of 14 focus group discussions, 16 key informant interviews (KIIs) and 14 in-depth interviews (IDIs). The respondents were purposively selected to achieve a heterogeneous sample who covered a range of possible characteristics that enabled detailed exploration and understanding of the central theme of the research questions. Purposive –criterion sampling technique was used to recruit IDI participants. The IDIs included HIV-positive or negative mothers with a child under 2 years of age and healthcare workers based at PMTCT clinics in health facilities. Key informants were selected based on their influence in the community, their knowledge on breastfeeding in terms of culture and other practices and their ability to mobilize the community to participate in the study. Triangulation of qualitative and quantitative data sources and diversification of study participants was meant to assure data quality. See Table 1.

**Table 1:** Description of sample

Interview type	Participants	Number
Focus group discussion	Young mothers	3
	Older mothers	3
	Fathers	3
	Community health volunteers	3
	Grandmothers	2
Key informant interviews	Community leaders	6
	Traditional birth attendants	2
	Religious leaders	3
	Health administrator	4
	Herbalist	1
In-depth interviews	Health care workers	4
	HIV positive mother with <2 year-old child	6
	Breastfeeding mother with < 2 year-old child	4
Total		**44**

### Data collection techniques and procedures

Individual face to face interviews were the core data collection method. An open-ended interview guide with a list of questions was used as the main tool for data collection. The focus of the interview guide was informed by WHO/UNICEF and MOH guidelines on infant feeding in the context of HIV. The moderator posed questions in a neutral manner and further probes followed the participants’ response.

The interviews were performed by the principal investigator at a convenient place either in a nearby school, church or facility. All FGDs were conducted by a team of three including a moderator, facilitator and the principal investigator. Individual IDIs, FGDs and KIIs were collected through verbatim tape transcripts, written summarized field notes of the interview and a debriefing form which were filled in at the end of each interview. Most interviews were conducted in the local language (Swahili) except for grandmothers, which were conducted in the local Kalenjin language with an experienced translator. The length of the interviews and FGDs ranged between 60 and 90 min.

### Data analysis

To establish barriers and facilitators influencing feeding practices for HIV exposed infants, themes were identified in advance based on the KII, IDI and FGD guide. A code book was developed using the interview guide as an initial basis for codes. Data collected were analyzed after the interview each day by the researcher assisted by data collectors in order to identify emerging themes that required further exploration in the subsequent interviews and to inform whether data saturation had been reached. Verbatim responses were transcribed and translated from Kiswahili into English. Transcribed data were analyzed using NVivo version 10.0. Additional codes emerging from common themes in the data were added.

### Data quality assurance

In order to maintain quality and credibility of the findings, all data collection tools were evaluated by professionals before data collection. The interview guide and probes were originally prepared in English by the lead researcher and then translated into the local language (Swahili). The guides were then reviewed, and feedback given by other members of the authorship team and the Kiswahili tools were sent to the locals and specialist language designers for back translation and rephrasing where necessary. Triangulation from multiple data sources were made and the diversity of the participants in terms of age, location and position was considered to enable deeper understanding of the research questions. Probes were used to obtain detailed information from participants.

### Informed consent

The study objectives, procedures benefits and risks were explained to the participants before recruitment. Informed consent by signature or thumbprint was sought from the participants before involving them in the study. If a participant was unable to read, the informed consent was read aloud to them. Each participant retained a copy of the signed or thumb printed informed consent form while the researcher retained one copy. Participation was voluntary and only mothers willing to participate were recruited into the study. All information provided to the investigator was considered confidential. The information was only for the study and would not be shared for any other purpose or projects.

## Results

### Facilitators of optimal breastfeeding

#### Counselling and support

Some participants reported that counseling at the prevention of mother-to-child transmission (PMTCT) clinic made women feel empowered and gave them confidence to adhere to infant feeding recommendations despite the community’s cultural norms and practices that dissuaded exclusive breastfeeding. As a result, some community members and mothers believed that if they followed the advice, the child would be healthy.

*“As long as you follow what you are advised the child will be fine and will not be infected with the virus even if you breastfeed.” (FGD_mother above 25_NubianVillage_Kamelilo).*

*“They normally say we Kalenjins believe in herbs to wash the child’s stomach so that the first black stool can come out, but for me since I attended PMTCT and received advice, I don’t believe and I did not give my child herbs and he never had stomach problems.” (IDI, HIV+ Positive mother with <2 E/Ravine CU.*

#### The desire to have a healthy baby

Some of the mothers had lost their child previously or had a positive child and they attributed the deaths to HIV/AIDs which they said was as a result of mixed feeding. They felt motivated to exclusively breastfeed as their current child was healthier, stronger and got ill less often.

*“I have two children one who is HIV positive and this one who is HIV negative. I think I transmitted the virus during breastfeeding to the first one since I gave the child herbs and they scratched the throat. But this one (who was exclusively breastfed) is very okay and healthy.” (IDI, HIV+ Positive mother with <2 E/Ravine CU)*.

#### Use of ARV medication

Some mothers reported that they had been taught that use of ARVs was effective in preventing HIV whilst breastfeeding.

*“In the past people never knew that you (HIV positive mother) can breastfeed the child but now people have been taught that you can breastfeed so long as you are taking the drugs then they are told that you should not give the child any other food other than breastmilk.” (FGDs, breastfeeding young mother with child <2, Solian CU).*

*“Those with HIV are given some tablets if they have to breastfeed so that the child can be safe.” (FGD Fathers mumberes).*

#### Health benefits of breastmilk

Mothers reported receiving information on the health benefits of breastmilk and reported this as a strong motivation to breastfeed. They believed that if they breastfed, the child would get less sick due to diarrhea and the immunity would be improved

*“It (breastfeeding) helps the child with digestion thus preventing issues like diarrhea and breastmilk is important because it increases a child’s immunity against diseases.” (IDI, HIV+ Positive mother with <2 Esageri CU)*.

### Barriers to breastfeeding

The following were identified as barriers to optimal breastfeeding:

#### Poor dissemination of policies related to infant feeding in the context of HIV

A substantial number said that there is poor dissemination and implementation of policies related to infant feeding in the context of HIV.

*“I can say some of the policies being brought on board are good but the implementation part is the problem. HIV positive mothers should be doing EBF for six months but you find back at home these people are not prepared because of lack of correct information.” (*KII SCCH Strategy KII).

Most health workers in the primary healthcare facilities did not have information on continuation of breast feeding beyond 6 months and they felt that dissemination of information did not cascade down to the lower levels.

*“Many disseminations are attended by top managers and they do not reach to some of us hence we sometimes lack the right information to give mothers.” (IDI Nurse Esageri CU).*

#### Knowledge gap on infant feeding recommendations in the context of HIV

The findings revealed that current guidelines on the recommendations for infant feeding in the context of HIV are not clear especially regarding the duration of EBF and continuation of breastfeeding. Most of the respondents said that a HIV positive mother should breastfeed for the first 6 months and then stop despite the national guidelines recommending exclusive breastfeeding for the first 6 months and thereafter continuation from 6 months in addition to optimal complementary feeding.

**“***A HIV positive mother should exclusively breastfeed for 6 months then stop completely.”* (*FGD_CHVs_Timboroa).*

*“A child is supposed to be breastfed for six months without being given any other food then he stops being breastfed completely.” (IDI, HIV+ Positive mother with <2 Esageri).*

*Okay for the HIV exposed babies we tell them to do EBF for the first six months then they stop the breastfeeding.” (IDI, Nurse Mercy Hospital E/Ravine).*

*“They are supposed to just breastfeed for six months not more than that and if they continue breastfeeding then they will transmit the virus.” (KII TBA Nubian Village Kamelilo E/Ravine CU).*

*They have been advised by doctors to avoid breastfeeding . . . but the child will finally die.” (FGD with grandmothers, Mumberes).*

*“When she got pregnant she was told not to breastfeed by the doctor but she breastfed so the child only stayed for two months and died. Later she gave birth again and she did not breastfeed and that child is older now that is what I have seen.” (FGD older mothers Timboroa).*

#### Fear of vertical transmission among HIV positive mothers, healthcare providers and fathers

Although knowledge had filtered through regarding the need to support mothers to continue breastfeeding to 1 year, some healthcare workers and mothers were still skeptical about continued breastfeeding for HIV positive mothers after 6 months, most prefer breastfeeding up to 6 months. The majority of the HIV positive mothers interviewed in this study had or were intending to exclusively breastfeed their babies up to 6 months as advised in the health facility but were not intending to continue breastfeeding as per the national guidelines which recommended continued breastfeeding up to 1 year. Some health workers and mothers expressed concerns and fear of transmitting infection due to continued breastfeeding after the introduction of complementary foods at 6 months. The overall attitude and perception of both mothers and health workers was that, contrary to the guidelines, breastfeeding must stop at 6 months with the introduction of any other foods.

*“To me yes, we have the guidelines recommending breastfeeding up to one year then stop but there is no need of having the mother breastfeed and infect the baby with the virus hence to me I still recommend stopping at 6 months until it is proven that it works.” (IDI, Nurse, Esageri CU).*

*“It is allowed for one year not more than that but that is a risk I would tell somebody it is better to stop at six months.” IDI, HIV+ Positive mother with <2 Esageri CU)*.

Some women mentioned that a lack of peer models or tangible examples demonstrating that feeding was safe had influenced their attitude and decision to stop breastfeeding.

*“Yes when the baby was born she was okay even at six months she said the child is still young so she breastfed for twelve months but when the child was tested she was found to be positive hence it’s safe to stop at 6 months.” (IDI HIV+_with <2 E/Ravine).*

To some mothers, they felt that immunity diminishes if the mother continues to breastfeed beyond 6 months and the child could be infected.

*“Breastfeed for six months because when you continue like for someone like me, the immunity goes down so it is important I breastfeed for six months then I stop so that I can take care of myself and take care of baby.” IDI HIV+_with <2 E/Ravine).*

There was of lack of support and attitude of community members that breastfeeding is not feasible for HIV positive mothers.

*“I don’t agree with the HIV positive women breastfeeding the child because the virus will be transmitted to the child. If there was an alternative it would be better.” (FGD young fathers with under 2 years Tugumoi).*

#### Inadequate counselling and support

Some mothers reported that health workers and managers did not have adequate time to counsel them on breastfeeding. Health workers and managers reported increased workload due to shortage of staff which caused a decline in the quality of counseling. This compromised the quality of counseling.

*“In the past when you were pregnant we would be told the way we should eat and take care of ourselves in the hospital but that is not there anymore just like the mothers have said they don’t teach in the facility they just test you and that is it . . . .” (FGD older Mothers Timboroa).*

*“The challenges is that at times the work load for health workers is a bit tough in maternity and those mothers can miss breastfeeding information. The flow of mothers is sometimes too much for the nurse to handle.” (KII_SubCounty_Nutritionist).*

Inadequate counseling was not only attributed to a lack of time and resources but was also reported by some as being due to lack of counseling skills.

*“At times we get the scenario that somebody who is not trained fully on PMTCT but only On the Job Training (OJT) is taken to that service entry point so at times to me the quality of PMTCT and counselling services may not be done to what it is supposed to be. The work load for the health workers is sometimes too much.” (KII SCCS_SCASCO).*

#### Misconception and misinformation that breastfeeding is for HIV positive mothers

Participants reported that EBF is the common choice for HIV positive mothers which is driven by the advice they receive from health workers. Exclusive breastfeeding was reported to be more highly promoted among the HIV positive mothers than among the rest of the community which was growing the stigma associated with EBF and being HIV positive. HIV positive mothers felt they were adhering to advice because of their HIV status.

*“The mother who is HIV negative breastfeeds the child throughout and they can give the baby food before six months since they don’t care but for us we have to be careful to protect the child.” (IDI HIV+ with child <2 Pilot E/Ravine CU).*

*“I heard from my wife and I saw her clinic card that she had tested for HIV so I asked her what happens to those who are HIV positive and she told me that when you hear a mother has been told to breastfeed for six months without giving the child anything else you know she is HIV positive.” (FGDs young fathers with child <2 Tugumoi CU).*

The presence of women who are HIV negative who were also breastfeeding but not practicing EBF posed a challenge for HIV positive mothers who were perceived to be HIV positive due to the fact that they were breastfeeding exclusively. This created stigma for the HIV positive mothers because exclusive breastfeeding in this community is associated with being HIV positive.

*“You have to breastfeed in secret so the other people are curious are you breastfeeding or not so they come to confirm because they ask why are you not giving the child other foods like other women.” (IDI HIV+ with child <2 E/Ravine CU).*

*“The first challenge is that when you just breastfeed and you don’t give anything else, the other women isolate you they do not want to mix with you also they do not want you to mix with them as they see like you will infect their children.” (IDI HIV+ with child <2 Esageri CU).*

Women who did not disclose their HIV status to their partners, mothers or mothers in law had challenges in EBF because they could not have an open dialogue about why they wanted to maintain exclusive breastfeeding. Some had to strategize their own ways in order to adhere to the recommendations from health workers as opposed to following the advice from the mother in-law.

*“My grandmother brought for me herbs and told me that they clean the child’s stomach so I told her to light the fire since at that time I am not allowed to go to the kitchen, but I can tell you the truth before God I poured it on the baby’s clothes until it was over.” (IDI HIV+ mother with child < 2 E/Ravine).*

#### Social pressure from family members and peers

Social pressure from mother in-laws and husbands to adhere to culture and traditions was reported which resulted in lack of control over breastfeeding practices. When mothers opted for the professional advice over the advice from the mother in-law and spouses it was interpreted as lack of respect from the daughter in-law and led to mistreatment and abandonment.

*“I was pregnant and I went to the clinic when it was five months so I was tested and I was told I am positive. When I went home I told my husband and he reacted negatively and he denied. My mother in law really humiliated me and she incited her son to leave me. She thought it was me who came with the disease and not her son.” (IDI HIV+ mother with child <2 E/Ravine).*

*“When I gave birth, I chose not to breastfeed but my mother in-law and my husband were insisting that I breastfeed. So in that state I became confused so I breastfed the baby for two weeks then they brought the formula which I started giving. But the health of the baby was not good he had diarrhea and vomiting and he later died. So after that one died I became pregnant again so with this pregnancy I had learnt that breastfeeding is good for the baby and my baby is negative (despite breastfeeding).” (HIV+ mother with child < 2 Torongo).*

#### Lack of male involvement

In the policies, it is recommended that a mother is accompanied to the clinic with his partner for testing and counselling for breastfeeding support. However, in this community, men rarely accompanied their wives to clinic.

*“but coming as a couple to PMTCT to be counseled together and tested or to be told of issues as pertains antenatal care clinic follow ups, men don’t come. For example, for the last three months out of many mothers who were seen in ANC only six were couples so the male involvement is very low.”* (KII\cr140824_004_SCC Strategy_KII).

#### Prelacteal feeds and perception of not having enough milk

Prelacteal feeding was widespread with feeds such as herbs, tonic water, plain water, water solution (glucose, salt and sugar), “sukaringuru” (molasses) solution and diluted milk being given as early as the first day of birth. It was given to feed the baby when it was perceived that “not enough milk is produced” after delivery, to relieve stomach upsets – when the child cried unnecessarily, to remove black stools and sometimes even based on advice by healthcare workers at health facilities.

*“When I was not giving him food he was having stomach upsets but when I gave him food his stomach was cleaned and the dirt that was in the stomach came out.” (FGD young mothers with child <2 Nubian Village Kamelilo E/Ravine CU).*

*“We were advised that the baby should be breastfed exclusively for six months but when we came back home the milk was not enough for the baby and I don’t want to lie I gave my child porridge.” (IDI HIV+ mother with child < 2 E/Ravine).*

Mothers defined EBF differently to mean excluding only solid foods from the breast. Some mothers were asked how they feed the baby and they reported that they give only breast milk but when probed further they confirmed they give water and glucose.

*“I give water every day like three spoons I thought you were asking about food.” (Older mothers with child <2 Timboroa).*

#### Employment and work

Employment was reported to be a challenge for breastfeeding mothers because mothers often spend a long time away from the infant and most workplaces do not offer lactation rooms or any support policies for breastfeeding.

*“She (breastfeeding mother) will leave at 8 and then go to work in someone's compound up to six in the evening until the boss comes back so you can imagine all those hours the baby has not breastfed but has been given other foods as they wait for the mother to come in the evening.” (IDI breastfeeding mother with child <2 Timboroa).*

Expressing breastmilk was considered as unnatural and unethical unless the mother was sick and it was a rare practice in this community thus limiting exclusive breastfeeding. There were concerns with issues of expressing and handling/preserving the milk, hygiene, and the perception that it was violating values instituted by God.

*“Unless the mother is sick or undergoes an operation expressing is not allowed in our community.” (FGD Grandmothers Solian CU).*

*“According to me I don’t agree with the expressing of milk because there are values which even when you look at the bible it is a personal thing because you cannot change you were created that way that when you give birth you breastfeed your baby so maybe according to me I oppose it or I will have gone against the values that are instituted by God.” (FGDs young fathers Arama CU).*

*“Some of the mothers do not wash their hands when they are expressing that milk then it will be contaminated so they should just breastfeed the child.” (FGD young fathers with child <2 Tugumoi).*

#### Food insecurity (access, availability, affordability, quality and quantity

The study showed that some women resorted to mixed feeding when they perceived themselves not to be able to provide adequate milk because of the poor quality of diet.

*“The mother could not breastfeed because she said she does not have food to eat hence the child could not have milk. We introduced the nutritionist Miriam to follow up the case. There is a big problem because the ARV drugs are not supposed to be taken on an empty stomach.” (KII SCCS SCASCO).*

## Discussion

### Facilitators influencing feeding practices for HIV exposed infants

This study aimed at exploring facilitators and barriers in regard to breastfeeding among HIV positive mothers by interviewing the HIV infected mothers themselves and other community members. The study has revealed that some women are motivated particularly to exclusively breastfeed for 6 months. Counselling services at the health facility, health status of the current child, use of ARV drugs and health benefits associated with breastmilk are some of the motivators identified. Some studies conducted in Kenya and Ethiopia have found similar results where counseling initiated prior to delivery and continued during the post-partum were the facilitators to EBF adherence [34–38].

HIV positive mothers desire to have a negative healthy baby and they will do all to ensure they protect her/him from HIV infection. In a review analysis study conducted in sub Saharan-Africa on infant feeding counselling to increase EBF by HIV-positive women, it was found that the desire to motivate child survival was key to EBF adherence [39]. There is recognition of the mothers on the role that ARVs play to reduce HIV infection. However, fear of infecting the baby through breastmilk is a threat to exclusive breastfeeding.

### Barriers to optimal feeding practices for HIV exposed infants

HIV women receive information on optimal breastfeeding practices. However, it is clear from the findings of this study that most women experienced barriers that hindered them from adhering to breastfeeding recommendations. These challenges could be linked to failure to follow optimal breastfeeding practices as reported by a study conducted in Uganda and South Africa [40, 41]. Heavy workload and shortage of staff has been identified by other studies as a hindrance to mothers getting adequate counselling. Studies conducted in China and Ethiopia found that health workers were constrained and did not have enough time to counsel mothers due to workload and staff shortage [37, 42]. Lack of infrastructure and shortage of trained personnel could be some of the factors that have made counseling difficult in these clinics.

Fear of vertical transmission of HIV from mother to child is common among the respondents in this study despite that they have received information on the benefits of breastfeeding the HIV exposed infants. This is in line with a study conducted in South Africa and Nigeria where the decision to stop breastfeeding despite having been counselled on continued breastfeeding was driven by fear [28, 43, 44]. Recent reviews conducted in a total of 27 studies on infant feeding counselling to increase EBF by HIV-positive women in sub Saharan-Africa, revealed that healthcare providers counselled mothers based on their own personal beliefs, discouraging EBF, often led mothers to make feeding decisions based on fear of HIV transmission, instead of the promotion of child survival [39].

The fear could have been prompted by frequent changes of global and Kenyan guidelines on infant feeding in the context of HIV where there is often a new version already available before the older ones are fully implemented in the country. Since the first detection of HIV virus, the recommendation evolved from support for replacement feeding, to recommending exclusive breastfeeding for the first 6 months then early cessation. Exclusive breastfeeding for the first 6 months and continued breastfeeding while adhering to ARV was adopted thereafter and later in 2016, and it was recommended that HIV-unexposed and –exposed infants exclusively breastfeed and continue breastfeeding to 2 years or beyond while adhering to ARV guidelines [7–10, 15, 45, 46]. This study took place in 2014 when the guideline was to exclusively breastfeed for the first 6 months and continue up to 1 year while adhering to ARV guidelines. This frequent change of guidelines could have resulted in mixed messages, confusion among healthcare workers and infected mothers when they are scared of the possibility of infecting the baby. The difficulties in accepting the concept of continued breastfeeding for the HIV exposed infants has led to non-compliance with national guidelines. Attitude of healthcare workers may lead to mis-advice of mothers hence lack of adherence to exclusive breastfeeding.

Stigma on HIV is high in this community. Promotion of EBF more heavily among HIV positive mothers than the general population, has created a perception among mothers and the community that it is an intervention meant for those who are positive. Women who continue to breastfeed their infants exclusively are closely monitored and are likely to be identified as HIV positive by community members because the local tradition is not to practice EBF to 6 months, and this is aligned with other studies in Nairobi, Botswana, South Africa, Zambia and Nigeria [28, 29, 47–49]. Traditionally in these communities mixed feeding is the norm and women who exclusively breastfeed will be confirmed HIV positive by their peers hence they avoid exclusive breastfeeding. These suspicions have made EBF difficult among HIV positive mothers which act as a barrier to exclusive breastfeeding.

Disclosure is very critical for HIV positive mothers to receive support on infant feeding. However, this is a problem in the current study area and the findings align with a study conducted in Kenya [50]. Disclosure may help a pregnant HIV positive woman to adhere to the infant feeding recommendations and enable her to take medication without fear. Despite the recommendation on EBF being common for all women in this community, the messages are not getting through for women who are not HIV positive suggesting a need for more general community counselling on the benefits of exclusive breastfeeding.

Infant feeding practices as seen in this study, do not only concern the mother but also her partner, relatives and community members as has been identified in several other studies conducted in Kenya, Ethiopia, Uganda, Morocco, and Thailand [34, 40, 51–54]. Social relation surrounding infant feeding play a major role in the woman’s ability to adhere to her choice to practice optimal breastfeeding behaviors regardless of her HIV status. The advice from healthcare providers meets the cultural barriers associated with the giving of prelacteal feeds or herbs, medicine and water. The mothers in law and husbands, who are key in supporting the mothers in the home environment, often give incorrect advice regarding infant feeding. This means that their knowledge regarding infant feeding becomes the predominant information that women receive in this community. This is common with previous studies conducted in South Africa, Morocco and Uganda [41, 52, 55]. Social pressure may extend beyond family to friends as documented in systematic review findings for sub-Saharan Africa countries [56, 57].

The perception of insufficient milk production may be a major hindrance to exclusive breastfeeding as reported in this study. When a child is crying it is perceived that the child is not satisfied with the breastmilk and needs other food. Other studies conducted in Kenya have documented similar findings [23, 58]. It is likely that these women lack information on how breastfeeding works including breastfeeding techniques. The mothers may have received inadequate skilled counselling to enable them to gain skills and competencies on proper breastfeeding techniques. This could justify why women in this study and previous studies conducted in Kenya, Mozambique and Nigeria complained of insufficient milk production and therefore resort to mixed feeding [28, 58–61].

The WHO guidelines recommend that women living with HIV should get support from health services to improve feeding practices with exclusive breastfeeding being the optimal goal [12, 62, 63]. Governments and local communities should therefore actively promote and implement services to create a supportive environment for mothers living with HIV to remain adherent to treatment and to breastfeed their infants in all settings: at work, at community centres, in health clinics, and in their homes.

## Conclusions

This research sheds light on the complexities of optimal breastfeeding and especially exclusive breastfeeding as an infant feeding practice among HIV positive mothers against the backdrop of socio-economic context, and cultural norms in the community. HIV positive women have to navigate and/or overcome barriers to EBF, breastfeeding, MTCT concerns, cultural barriers, stigma, societal and family pressures, to make the best and most feasible feeding choice for their infants. This study reveals evidence for building up a strong continuum of care after women deliver in the hospital for supporting both HIV positive and negative mothers from pregnancy up to 2 years and beyond especially at the community level. Therefore, support for all women regardless of their status with the same messages and including influential family members and communities would improve knowledge on the potentially harmful effects of following cultural traditions to mix feed from an early age and other barriers in the context of HIV. Community based counsellors who work with mothers as well as influencing family members, would be key in supporting women to achieve decisions that they make to exclusive breastfeeding more successfully than is currently happening in this community. Kenya is currently engaged in rolling out a community based strategy to support breastfeeding through its endorsement of the Baby Friendly Community Initiative [64]. Further, work is now needed in the counties that have introduced the BFCI initiative to establish whether this strategy has improved breastfeeding practices among HIV positive women, and to understand whether there remain unmet needs for this vulnerable group.

Recommendations that would contribute to supporting HIV positive mothers to adhere to breastfeeding include, integrating family support into the PMTCT program, having an exclusive breastfeeding policy for all women so that HIV positive women are not stigmatized, building the capacity for the healthcare professionals including frequent update of information on HIV and breastfeeding.

## Data Availability

The data are available from the African Population and Health Research Center (APHRC) upon reasonable request and with permission of APHRC.

## Abbreviations

APHRC: African Population and Health Research Center
ARVs: Antiretrovirals
BFCI: Baby Friendly Community Initiative
eMTCT: Elimination of Mother to Child Transmission
EBF: Exclusive breastfeeding
KEMRI: Kenya Medical Research Institute
MIYCN: Maternal Infant and Young Child Nutrition
MTCT: Mother to Child Transmission
NACOSTI: National Commission for Science, Technology and Innovations
SCHMT: Sub-County Health Management Team
TBA: Traditional Birth Attendants
